# Development and validation of machine learning nomograms for predicting mortality after cardiac valve surgery

**DOI:** 10.3389/fmed.2026.1779140

**Published:** 2026-03-27

**Authors:** Mateus Tamba N'dende Macho, Yi Song, Aojie Wei, Xi Zhao, Athukoralage Divasara Nethmini, Socheat Cheam, Hang Xing, Leiya Fu, Zhengyang Han, Xiangnan Li, Zhikun Fu, Qinglin Fu

**Affiliations:** 1 Department of Cardiovascular Surgery, The First Affiliated Hospital of Zhengzhou University, Zhengzhou, China; 2 Department of Ultrasound, The First Affiliated Hospital of Zhengzhou University, Zhengzhou, China; 3 Department of Cardiovascular Surgery, The 7th People's Hospital of Zhengzhou, Zhengzhou, China; 4 Department of Pediatrics, Women and Infants Hospital of Rhode Island, The Alpert Medical School of Brown University, Providence, RI, United States; 5 Department of Infectious Diseases, The First Affiliated Hospital of Zhengzhou University, Zhengzhou, Henan, China; 6 Department of Thoracic Surgery, The First Affiliated Hospital of Zhengzhou University, Zhengzhou, China

**Keywords:** Boruta algorithm, cardiac valve surgery, EuroSCORE II, machine learning, mortality, nomogram, risk prediction

## Abstract

**Objective:**

To develop and validate machine learning (ML) models for predicting mortality after heart valve surgery and compare their performance to the conventional EuroSCORE II, with the final goal of creating clinically accessible and easy-to-use nomograms.

**Methods:**

This multicenter, retrospective cohort study included 935 adult patients who underwent heart valve surgery. All-cause mortality at in-hospital, 30-day, and 365-day post-operative intervals were the primary outcomes. The Boruta algorithm was employed for feature selection. Five models, Logistic Regression, XGBoost, Random Forest, Extra Trees, and EuroSCORE II (as a benchmark), were developed on a 70% training set and validated on a 30% hold-out test set. Model performance was evaluated using the Area Under the Receiver Operating Characteristic Curve (ROC AUC), sensitivity, and specificity. The best-performing model for each endpoint was converted into a nomogram.

**Results:**

Machine learning models demonstrated strong discriminative performance across all endpoints. For in-hospital mortality, the Extra Trees model achieved the highest discrimination (ROC-AUC 0.858). For 30-day mortality, Logistic Regression showed the best performance (ROC-AUC 0.800), substantially exceeding EuroSCORE II (ROC-AUC 0.610). For 365-day mortality, predictive performance was comparable across models, with EuroSCORE II demonstrating similar discrimination (ROC-AUC 0.787). Key predictive features consistently included age and biomarkers reflecting cardiac stress, renal function, and hepatic function. The derived nomograms exhibited good discrimination and calibration in both internal and external validation cohorts.

**Conclusion:**

Machine learning models, particularly ensemble approaches, improved short-term mortality prediction following valve surgery compared with EuroSCORE II. The developed nomograms provide a practical and interpretable tool for individualized perioperative risk stratification.

## Introduction

Valvular heart disease (VHD) represents a major and growing healthcare concern, with a projected prevalence of approximately 2.5% in the general population, increasing to over 12% among individuals aged 75 years or older (1, 2). For patients with severe, symptomatic VHD, cardiac valve surgery remains the definitive treatment, with several hundred thousand procedures performed annually to improve survival and reduce morbidity (3).

Although there have been major advances in surgical methods and care, there are nonetheless inherent risks in conducting valve surgery. Mortality rates for in-hospital isolated VHD surgery have been estimated to vary from 2 to 6% based on associated comorbidities (4). Accurate pre-operative risk stratification is therefore paramount for informed shared decision-making, realistic patient and family counseling, and guiding clinical resource allocation towards high-risk individuals who may benefit from intensified monitoring and management (5).

Currently, preoperative risk evaluation remains largely driven by validated risk predictors like the European System for Cardiac Operative Risk Evaluation (EuroSCORE) II and the Society of Thoracic Surgeons (STS) Predicted Risk of Mortality (6, 7). These logistic regression-based risk predictors rely on a few selected clinical predictors to yield a predictive risk value. Their accuracy has, however, proved to be less optimal in particular groups, for example, in patients undergoing valve operations, with generally low discriminative power (AUC 0.70–0.80) and inaccurate calibration, especially in these particular groups (8, 9). This could be due to their failure to adequately model complex interactions among many predictive preoperative clinical variables.

Machine Learning (ML), a core component of Artificial Intelligence (AI) subfield, represents an efficient paradigm to deal with these challenges. While regular statistical models require raw data to fit pre-defined hypotheses, ML algorithms have the capacity to extract complex patterns from large data sets independently (10). Various applications in cardiovascular diseases, like heart failure, atrial fibrillation, and cardiac deaths post-coronary syndromes, showed improved predictive accuracy with ML (11, 12). Its property to capture multiple interactions between numerous variables, like demographics, clinical, laboratory, and echo information, proves to be highly advantageous in accurately estimating mortality post-cardiac surgery.

Although some studies have begun to explore ML for cardiac surgery outcomes, there is still a need for a thorough assessment. Most studies have examined results at a specific time point or have not included comparison with modern risk predictions, such as EuroSCORE II, for several different timescales, such as in-hospital, 30-day, and 365-day survival (13). Additionally, “black box” predictions by complex algorithms in some ML analyses could make such predictions difficult to implement in clinical practice. It has become necessary not only to reveal which features or algorithms have predictive value, but to transform those algorithms into something useful for decision-making at the “bedside” (14).

To fill these gaps, our study aimed at identifying the most robust predictive features by the use of the Boruta algorithm, to systematically train and compare the performance of multiple ML models against the traditional EuroSCORE II, and finally, to create and present simple nomograms depending on the best-performing ML model for each clinical time-point, which facilitates individualized risk assessment in clinical practice.

## Methods

### Study design and population

This was a multicenter, retrospective cohort study, and it was carried out at First Affiliated Hospital of Zhengzhou University. The protocol of this research was reviewed and approved by the Institutional Review Board at our institution, and we proceeded without seeking consent due to its retrospective nature. A total of 803 consecutive adult patients who underwent cardiac valve surgery between May 3, 2019, and January 10, 2024, were included. Eligible patients were those who received isolated or combined mitral, aortic, or tricuspid valve repair or replacement. Individuals undergoing concomitant major non-valvular cardiac procedures such as coronary artery bypass grafting, Bentall surgery, aortic dissection repair, or the Morrow procedure were excluded. Patients with intraoperative mortality, missing essential clinical information, or uncertain 30-day or 1-year survival outcomes were also excluded. To evaluate the generalizability and robustness of the predictive models, an independent external validation cohort (*n* = 132) was additionally obtained from The 7th People’s Hospital of Zhengzhou. The same inclusion and exclusion criteria were applied to this cohort to ensure methodological consistency and comparability across datasets.

### Data collection and preprocessing

Candidate predictors were primarily restricted to preoperative variables to ensure clinical applicability at the time of surgical decision-making. Preoperative medication use was not incorporated due to heterogeneity in clinical documentation and the potential for confounding by indication, which may bias treatment-effect interpretations in retrospective datasets. Duration of surgery and surgery approach were retained as a pragmatic proxy for procedural complexity rather than a mechanistic intraoperative variable. Within this framework, the extracted information encompassed demographic characteristics, comorbidities, vital signs, comprehensive laboratory assessments (including BNP, Troponin, and renal and liver function markers), echocardiographic measurements, surgical information, and EuroSCORE II. All variables were obtained from the electronic medical record systems of the participating institutions. The main outcome measures included all-cause mortalities at three points post-operatively: in-hospital, at 30 days, and at 365 days post-operative. To create balanced subsets, the dataset underwent stratified random sampling, making a training set comprising 70% of the data and an internal test set containing the other 30%.

### Feature selection and model development

**Figure 1:**
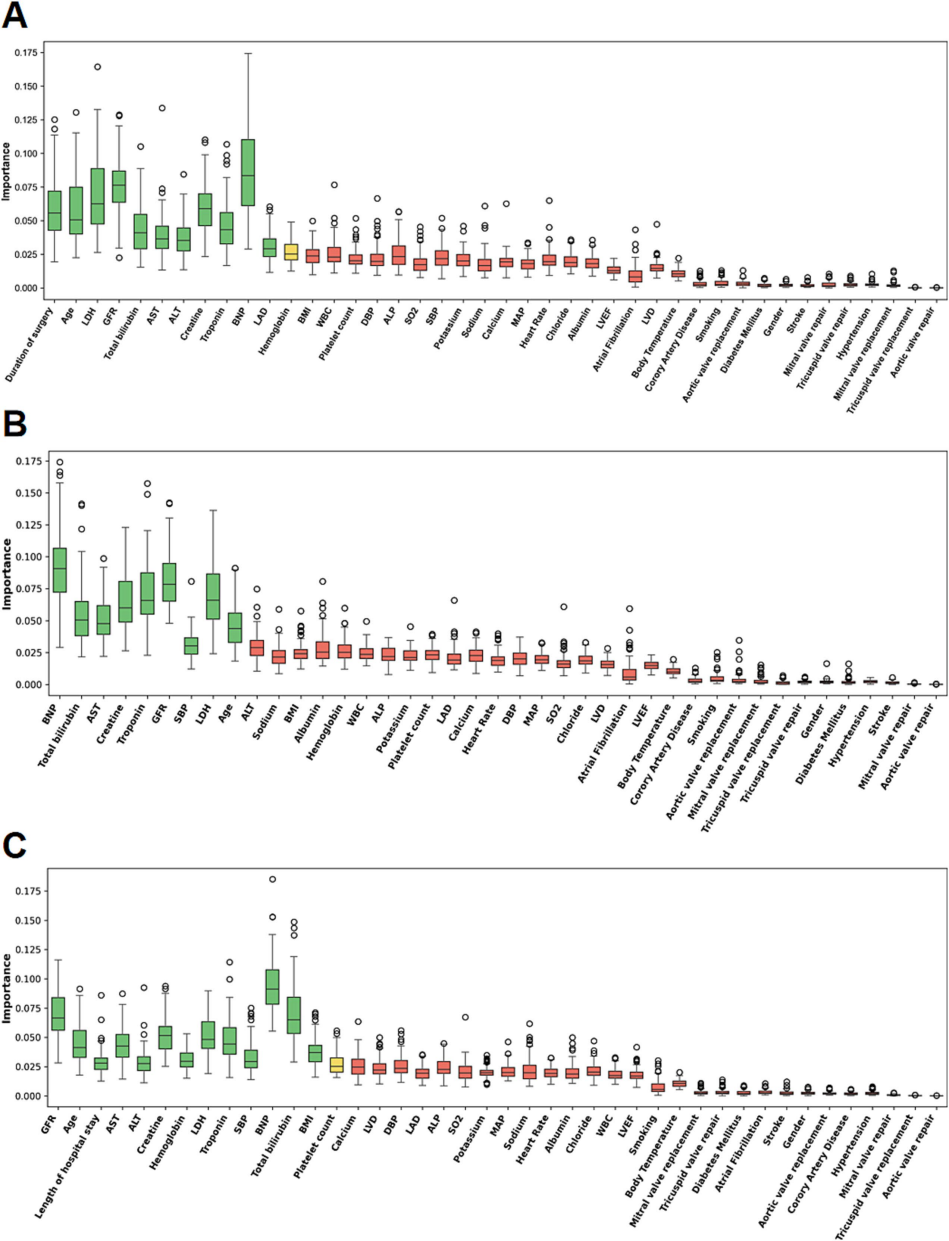
Boruta feature-selection results for three mortality outcomes. **(A)** Important predictors selected for in-hospital mortality. **(B)** Important predictors selected for 30-day mortality. **(C)** Important predictors selected for 365-day mortality.

To find the most significant predictors for each endpoint, we used the Boruta algorithm on training set, which is a robust wrapper algorithm incorporating a Random Forest classifier, analyzing feature significance by comparing it with randomly generated shadow attributes. The set of confirmed significant features for each time interval is displayed in Figure 1. After we identified this, we progressed with implementing five different models for predictions: Logistic Regression, XGBoost, Random Forest, Extra Trees, and EuroSCORE II as the clinical benchmark model. The ML models were subsequently trained using stratified 5-fold cross-validation on the training cohort, with hyperparameters optimized via grid search, targeting maximization of the ROC-AUC performance. Given the threshold dependency of classification metrics, the optimal classification threshold was determined using the Youden index.

### Model evaluation and clinical translation

**Figure 2:**
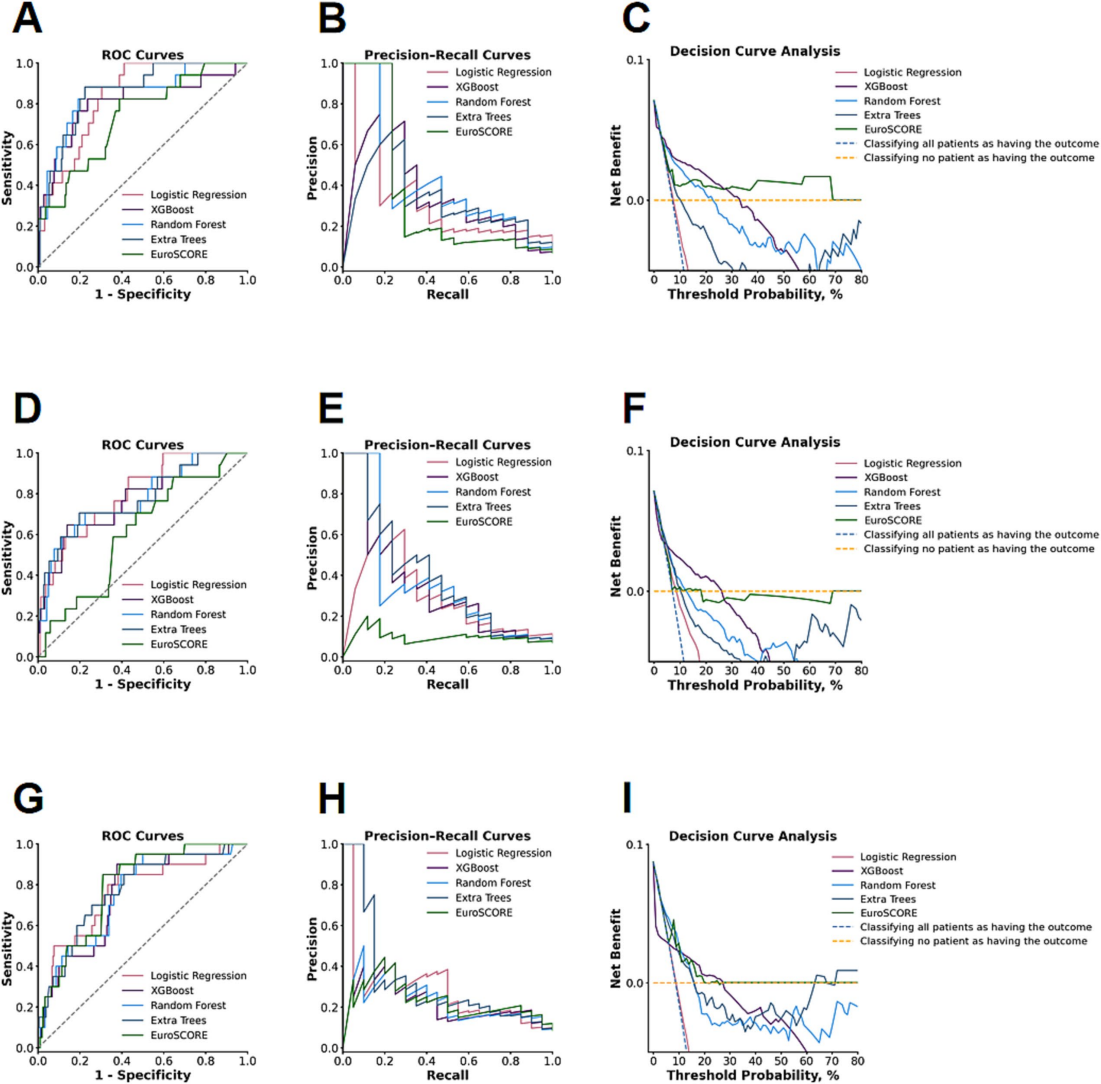
Performance comparison of five machine learning models (Logistic Regression, XGBoost, Random Forest, Extra Trees, and EuroSCORE II) across three mortality outcomes. **(A–C)** In-hospital mortality: ROC curves **(A)**, precision–recall curves **(B)**, and decision-curve analysis (DCA) **(C)**. **(D–F)** Thirty-day mortality: ROC curves **(D)**, precision–recall curves **(E)**, and DCA curves **(F)**. **(G–I)** Three-hundred-sixty-five–day mortality: ROC curves **(G)**, precision–recall curves **(H)**, and DCA curves **(I)**.

**Figure 3:**
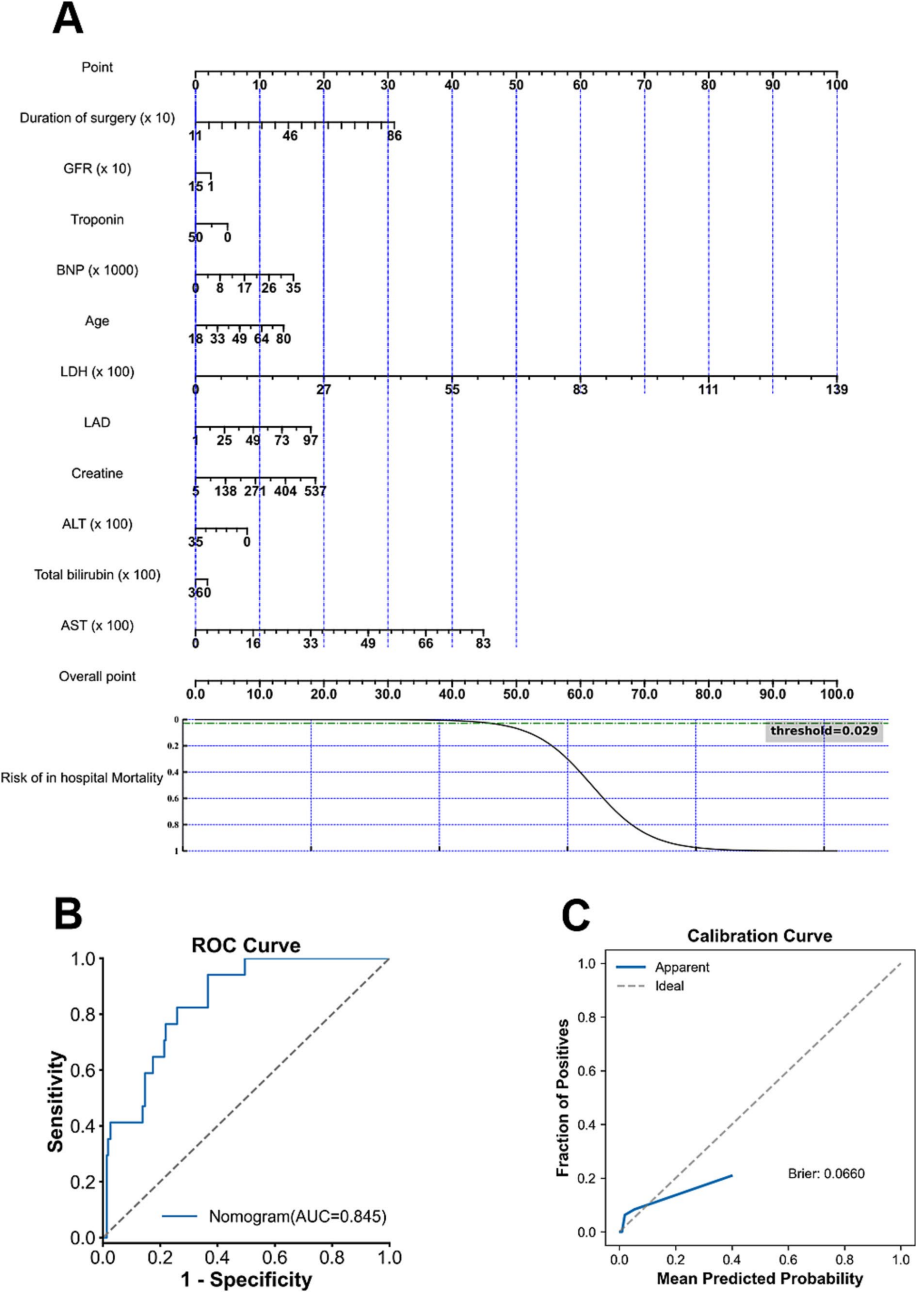
Nomogram development and performance evaluation for in-hospital mortality. **(A)** Final nomogram constructed using the selected predictors. **(B)** ROC curve demonstrating the discrimination performance of the nomogram. **(C)** Calibration curve showing agreement between predicted and observed risks.

**Figure 4:**
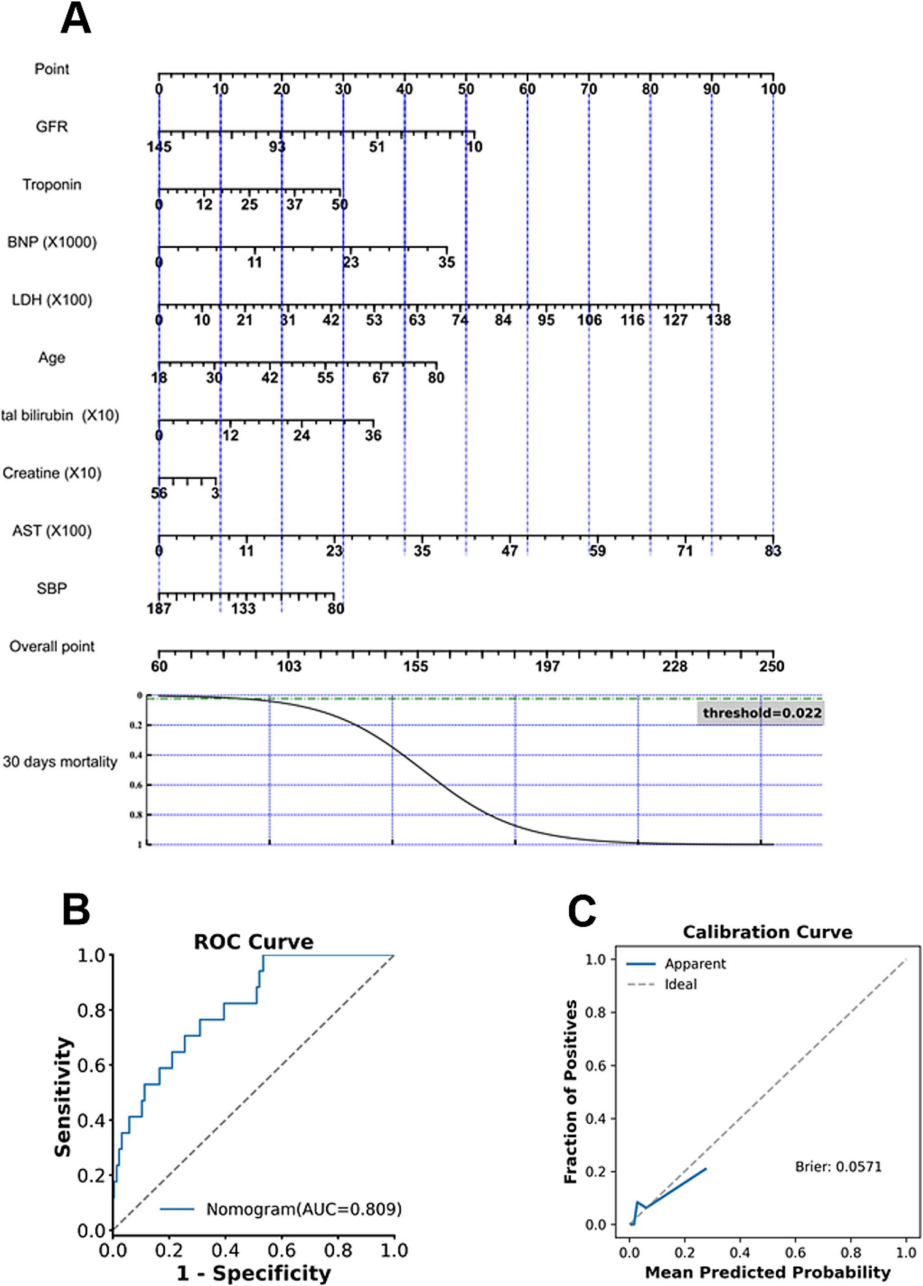
Nomogram development and performance evaluation for 30-day mortality. **(A)** Final nomogram constructed using the selected predictors. **(B)** ROC curve demonstrating the discrimination performance of the nomogram. **(C)** Calibration curve showing agreement between predicted and observed risks.

**Figure 5:**
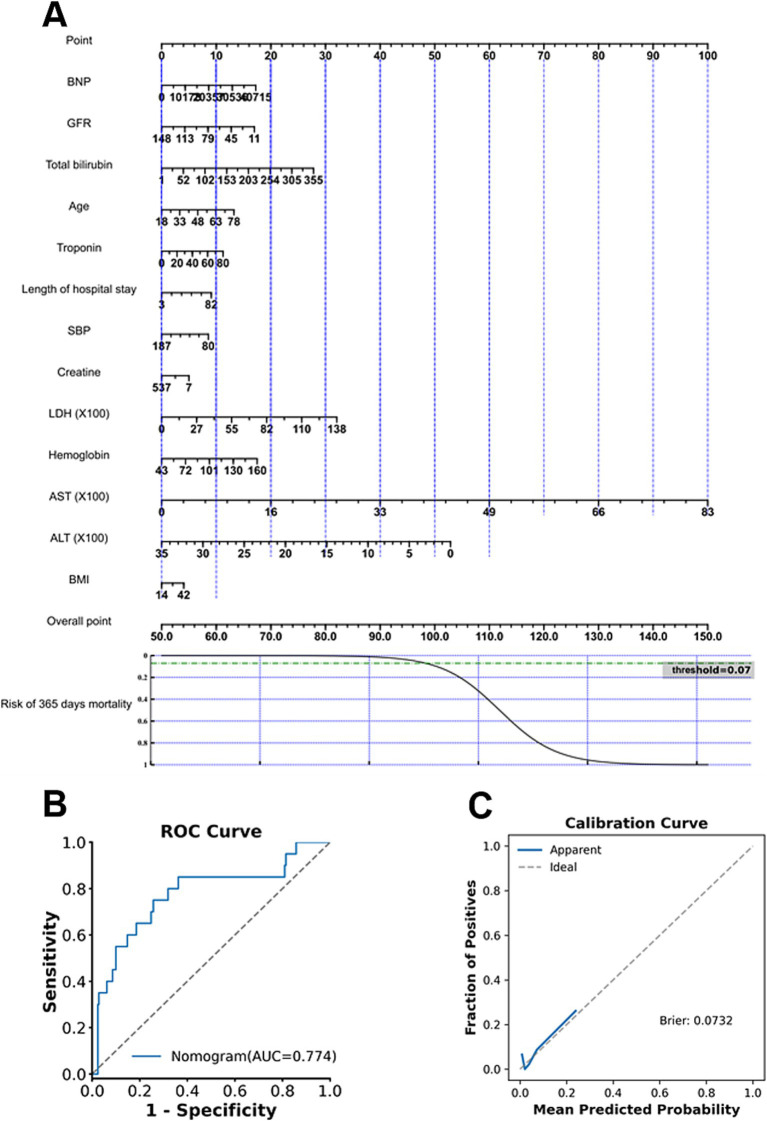
Nomogram development and performance evaluation for 365-day mortality. **(A)** Final nomogram constructed using the selected predictors. **(B)** ROC curve demonstrating the discrimination performance of the nomogram. **(C)** Calibration curve showing agreement between predicted and observed risks.

All models were tested on the hold-out test set. The performance was evaluated by Accuracy, Precision, Sensitivity, Specificity, and ROC AUC, with their respective 95% confidence intervals. The DeLong test was employed to make statistical comparisons of ROC AUC values between models. To further analyze and assess performance concerning class imbalance and clinical net benefits, respectively, Precision-Recall curves and Decision Curve Analysis were also employed (Figure 2). To enhance clinical interpretability and practicability, models demonstrating optimal discrimination performance were selected for each time horizon. Feature selection guided by the optimal ML models was followed by refinement using the FMA algorithm. Predictors identified as clinically relevant were subsequently incorporated into a Logistic Regression framework for development of interpretable nomograms (Figures 3–5). The discriminative power and excellent performance concerning each final nomogram constructed above were then evaluated by its ROC Curve and Calibration Plot, respectively.

### Statistical analysis

The Mann–Whitney U test and Chi-squared test or Fischer’s exact test were used to compare the baseline characteristics of survivors and non-survivors. A *p*-value of < 0.05 was considered significant. All statistical analyses were conducted using Python with standard libraries, such as scikit-learn, pandas, numpy, and statsmodels.

## Results

### Study population and baseline characteristics

A cumulative total of 803 patients who had heart valve surgery were included in this analysis. The incidence of all-cause mortalities was 6.8% (55 patients) at in-hospital, 7.4% at 30 days post-discharge (57 patients), and 8.9% at 365 days post-discharge (68 patients). The various baseline characteristics, stratified according to survival groups at each instance, can be found in Tables 1–3. It must be noted that all three groups overlap, with 30-day survivors including in-hospital survivors, and all survivors at 365 days.

**Table 1:** Baseline demographic, clinical, and laboratory characteristics of patients stratified by in-hospital mortality.

Variable	Alive (*N* = 748)	Death (*N* = 55)	*P*-value
Gender, *n* (%)			
Male	439 (58.7%)	30 (54.5%)	0.65
Female	309 (41.3%)	25 (45.5%)	
Age, Median (IQR)	50.00 (40.00–58.00)	61.00 (51.00–68.00)	<0.01
Hypertension, *n* (%)	168 (22.5%)	10 (18.2%)	0.57
Corory Artery Disease, *n* (%)	85 (11.4%)	12 (21.8%)	0.04
Atrial Fibrillation, *n* (%)	164 (21.9%)	24 (43.6%)	<0.01
Diabetes Mellitus, *n* (%)	28 (3.7%)	3 (5.5%)	0.78
Stroke, *n* (%)	84 (11.2%)	10 (18.2%)	0.18
Smoking, *n* (%)	219 (29.3%)	7 (12.7%)	0.01
Body Temperature, Median (IQR)	36.50 (36.30–36.60)	36.50 (36.30–36.65)	0.34
Heart Rate, Median (IQR)	80.00 (76.00–87.00)	80.00 (72.00–90.50)	0.66
SBP, Median (IQR)	122.00 (115.00–130.00)	116.00 (100.00–130.00)	0.02
DBP, median (IQR)	72.00 (65.00–80.00)	74.00 (60.00–79.50)	0.25
MAP, Median (IQR)	50.00 (40.00–58.00)	47.00 (36.50–55.50)	0.10
BMI, Median (IQR)	23.91 (21.48–26.10)	23.91 (21.25–24.27)	0.44
LVEF, Median (IQR)	61.00 (56.00–63.00)	60.00 (53.00–63.00)	0.15
LAD, Median (IQR)	45.00 (40.00–52.00)	48.00 (41.50–57.00)	0.02
LVD, Median (IQR)	54.00 (47.00–62.00)	51.00 (47.00–61.00)	0.71
BNP, Median (IQR)	1290.00 (484.75–3044.28)	3680.00 (2383.50–10748.60)	<0.01
Troponin, Median (IQR)	0.28 (0.02–1.64)	1.47 (0.18–5.15)	<0.01
WBC, Median (IQR)	13.73 (10.13–17.17)	13.10 (11.05–16.74)	0.72
Hemoglobin, Median (IQR)	105.00 (94.73–116.00)	102.00 (90.00–116.00)	0.28
Platelet count, Median (IQR)	132.00 (103.00–176.00)	111.00 (80.00–155.00)	<0.01
Potassium, Median (IQR)	4.11 (3.70–4.50)	4.20 (3.90–4.74)	0.09
Sodium, Median (IQR)	141.00 (139.00–144.00)	143.00 (138.00–148.00)	0.06
Chloride, Median (IQR)	105.00 (102.00–108.00)	104.00 (101.00–108.00)	0.18
Calcium, Median (IQR)	1.17 (1.10–1.39)	1.18 (1.12–2.09)	0.22
SO2, Median (IQR)	99.00 (98.00–100.00)	99.00 (97.45–99.35)	0.08
Creatine, Median (IQR)	78.00 (65.00–97.00)	104.00 (80.50–155.50)	<0.01
ALT, Median (IQR)	21.00 (15.00–31.00)	29.00 (21.50–90.00)	<0.01
AST, Median (IQR)	63.00 (42.00–90.00)	94.00 (61.00–175.00)	<0.01
ALP, Median (IQR)	58.00 (49.00–69.00)	63.00 (49.00–77.00)	0.16
Albumin, Median (IQR)	37.40 (33.60–40.80)	34.90 (33.20–38.80)	0.01
Total bilirubin, Median (IQR)	17.20 (11.70–26.10)	31.90 (13.30–55.25)	<0.01
GFR, median (IQR)	88.78 (70.26–105.46)	59.59 (34.62–81.48)	<0.01
LDH, median (IQR)	407.00 (244.00–467.27)	543.00 (465.63–971.50)	<0.01
Mitral valve replacement, *n* (%)	494 (66.0%)	45 (81.8%)	0.02
Mitral valve repair, *n* (%)	26 (3.5%)	2 (3.6%)	1.00
Tricuspid valve replacement, *n* (%)	8 (1.1%)	1 (1.8%)	1.00
Tricuspid valve repair, *n* (%)	323 (43.2%)	31 (56.4%)	0.08
Aortic valve replacement, *n* (%)	369 (49.3%)	18 (32.7%)	0.03
Aortic valve repair, *n* (%)	4 (0.5%)	0 (0.0%)	1.00
Duration of surgery, Median (IQR)	225.00 (186.00–280.00)	270.00 (198.50–413.50)	<0.01
EuroSCORE II, Median (IQR)	2.33 (1.38–3.96)	5.29 (3.08–11.37)	<0.01

**Table 2:** Baseline demographic, clinical, and laboratory characteristics of patients stratified by 30-day mortality.

Variable	Alive (*N* = 718)	Death (*N* = 57)	*P*-value
Gender, *n* (%)			
Male	433 (60.3%)	31 (54.4%)	0.64
Female	285 (39.7%)	26 (45.6%)	
Age, Median (IQR)	50.00 (40.00–58.00)	61.00 (49.00–68.00)	<0.01
Hypertension, *n* (%)	165 (22.3%)	11 (19.3%)	0.72
Corory Artery Disease, *n* (%)	83 (11.2%)	13 (22.8%)	0.02
Atrial Fibrillation, *n* (%)	162 (21.9%)	25 (43.9%)	<0.01
Diabetes Mellitus, *n* (%)	27 (3.6%)	4 (7.0%)	0.36
Stroke, *n* (%)	81 (10.9%)	11 (19.3%)	0.09
Smoking, *n* (%)	218 (29.5%)	8 (14.0%)	0.02
Body Temperature, Median (IQR)	36.50 (36.30–36.60)	36.50 (36.30–36.60)	0.41
Heart Rate, Median (IQR)	80.00 (76.00–87.00)	80.00 (72.00–89.00)	0.60
SBP, Median (IQR)	122.00 (115.00–130.00)	116.00 (100.00–130.00)	0.02
DBP, Median (IQR)	72.00 (65.00–80.00)	74.00 (60.00–80.00)	0.27
MAP, Median (IQR)	50.00 (40.00–58.00)	47.00 (37.00–55.00)	0.08
BMI, Median (IQR)	23.91 (21.48–26.10)	23.91 (20.72–24.09)	0.27
LVEF, Median (IQR)	61.00 (56.00–63.00)	60.00 (53.00–63.00)	0.09
LAD, Median (IQR)	45.00 (40.00–52.00)	47.00 (42.00–55.00)	0.02
LVD, Median (IQR)	54.00 (47.00–62.00)	51.00 (47.00–60.00)	0.56
BNP, Median (IQR)	1287.00 (476.03–3044.28)	3680.00 (2305.00–11937.20)	<0.01
Troponin, Median (IQR)	0.28 (0.02–1.64)	1.64 (0.18–5.22)	<0.01
WBC, Median (IQR)	13.71 (10.13–17.16)	13.10 (11.06–16.78)	0.81
Hemoglobin, Median (IQR)	105.00 (94.97–116.00)	102.00 (90.00–118.00)	0.33
Platelet count, Median (IQR)	132.00 (103.00–176.00)	111.00 (81.00–153.00)	<0.01
Potassium, Median (IQR)	4.11 (3.70–4.50)	4.20 (3.90–4.68)	0.12
Sodium, Median (IQR)	141.00 (139.00–144.00)	143.00 (138.00–148.00)	0.07
Chloride, Median (IQR)	105.00 (102.00–108.00)	104.00 (101.00–108.10)	0.28
Calcium, Median (IQR)	1.17 (1.10–1.38)	1.18 (1.12–2.06)	0.20
SO2, Median (IQR)	99.00 (98.00–100.00)	99.00 (97.40–99.40)	0.06
Creatine, Median (IQR)	78.00 (65.00–96.25)	104.00 (78.00–152.00)	<0.01
ALT, Median (IQR)	21.00 (15.00–31.00)	29.00 (21.00–89.00)	<0.01
AST, Median (IQR)	62.50 (41.75–90.00)	94.00 (64.00–177.00)	<0.01
ALP, Median (IQR)	58.00 (49.00–69.00)	63.00 (49.00–77.00)	0.12
Albumin, Median (IQR)	37.40 (33.60–40.80)	34.90 (33.20–38.90)	0.02
Total bilirubin, Median (IQR)	17.15 (11.68–25.90)	32.00 (14.70–53.20)	<0.01
GFR, Median (IQR)	89.08 (70.26–105.53)	59.66 (34.99–82.05)	<0.01
LDH, Median (IQR)	407.50 (244.75–467.27)	493.00 (435.00–955.00)	<0.01
Mitral valve replacement, *n* (%)	487 (65.8%)	47 (82.5%)	0.02
Mitral valve repair, *n* (%)	26 (3.5%)	2 (3.5%)	1.00
Tricuspid valve replacement, *n* (%)	8 (1.1%)	1 (1.8%)	1.00
Tricuspid valve repair, *n* (%)	317 (42.8%)	33 (57.9%)	0.04
Aortic valve replacement, *n* (%)	367 (49.6%)	19 (33.3%)	0.03
Aortic valve repair, *n* (%)	4 (0.5%)	0 (0.0%)	1.00
EuroSCORE II, Median (IQR)	2.31 (1.35–3.95)	3.35 (3.17–5.79)	<0.01

**Table 3:** Baseline demographic, clinical, and laboratory characteristics of patients stratified by 365-day mortality.

Variable	Alive (*N* = 698)	Death (*N* = 68)	*P*-value
Gender, *n* (%)			
Male	409 (58.6%)	37 (54.4%)	0.59
Female	289 (41.4%)	31 (45.6%)	
Age, Median (IQR)	50.00 (40.00–58.00)	59.50 (48.00–68.00)	<0.01
Length of hospital stay, Median (IQR)	22.00 (18.00–30.00)	26.00 (15.00–39.00)	0.49
Hypertension, *n* (%)	158 (22.6%)	15 (22.1%)	1.00
Corory Artery Disease, *n* (%)	78 (11.2%)	17 (25.0%)	<0.01
Atrial Fibrillation, *n* (%)	149 (21.3%)	28 (41.2%)	<0.01
Diabetes Mellitus, *n* (%)	27 (3.9%)	4 (5.9%)	0.63
Stroke, *n* (%)	73 (10.5%)	13 (19.1%)	0.05
Smoking, *n* (%)	205 (29.4%)	9 (13.2%)	<0.01
Body Temperature, Median (IQR)	36.50 (36.30–36.60)	36.50 (36.30–36.60)	0.80
Heart Rate, Median (IQR)	80.00 (76.00–87.00)	80.50 (72.00–88.25)	0.79
SBP, Median (IQR)	122.09 (115.00–130.00)	115.50 (101.50–133.00)	<0.01
DBP, Median (IQR)	72.00 (65.00–80.00)	68.50 (62.00–80.00)	0.14
MAP, Median (IQR)	50.00 (41.00–58.00)	46.50 (36.00–55.25)	0.03
BMI, Median (IQR)	23.91 (21.48–26.19)	23.91 (21.09–24.18)	0.26
LVEF, Median (IQR)	61.00 (56.00–63.00)	58.32 (51.75–62.00)	<0.01
LAD, Median (IQR)	45.00 (40.00–51.75)	47.00 (40.75–54.25)	0.03
LVD, Median (IQR)	54.00 (47.00–62.00)	53.50 (47.00–60.50)	0.62
BNP, Median (IQR)	1283.05 (465.00–3044.28)	3638.00 (1954.37–10154.30)	<0.01
Troponin, Median (IQR)	0.27 (0.02–1.64)	1.42 (0.07–4.84)	<0.01
WBC, Median (IQR)	13.65 (10.20–17.16)	13.08 (10.11–16.72)	0.57
Hemoglobin, Median (IQR)	105.00 (95.00–116.00)	102.00 (90.00–116.50)	0.14
Platelet count, Median (IQR)	133.50 (104.00–176.00)	110.50 (78.00–151.25)	<0.01
Potassium, Median (IQR)	4.11 (3.73–4.50)	4.20 (3.90–4.67)	0.13
Sodium, Median (IQR)	141.00 (139.00–144.00)	143.00 (138.75–147.25)	0.02
Chloride, Median (IQR)	105.00 (102.00–108.00)	104.85 (101.92–108.93)	0.96
Calcium, Median (IQR)	1.17 (1.10–1.38)	1.17 (1.12–2.05)	0.32
SO2, Median (IQR)	99.00 (98.00–100.00)	99.00 (97.30–99.53)	0.14
Creatine, Median (IQR)	78.00 (65.00–97.00)	101.50 (75.50–150.25)	<0.01
ALT, Median (IQR)	22.00 (15.00–31.00)	28.50 (19.75–52.50)	<0.01
AST, Median (IQR)	63.00 (41.00–90.75)	91.00 (61.00–165.25)	<0.01
ALP, Median (IQR)	58.00 (49.00–69.00)	64.00 (51.00–77.25)	0.06
Albumin, Median (IQR)	37.50 (33.62–40.90)	34.80 (33.20–38.75)	<0.01
Total bilirubin, Median (IQR)	16.90 (11.60–25.80)	29.90 (14.00–52.47)	<0.01
GFR, Median (IQR)	89.26 (70.36–105.46)	61.21 (40.35–85.77)	<0.01
LDH, Median (IQR)	407.00 (244.25–467.27)	467.27 (327.25–898.25)	<0.01
Mitral valve replacement, *n* (%)	457 (65.5%)	55 (80.9%)	0.01
Mitral valve repair, *n* (%)	25 (3.6%)	2 (2.9%)	1.00
Tricuspid valve replacement, *n* (%)	8 (1.1%)	1 (1.5%)	1.00
Tricuspid valve repair, *n* (%)	294 (42.1%)	40 (58.8%)	0.01
Aortic valve replacement, *n* (%)	346 (49.6%)	26 (38.2%)	0.10
Aortic valve repair, *n* (%)	4 (0.6%)	0 (0.0%)	1.00
EuroSCORE II, Median (IQR)	2.20 (1.34–3.96)	3.35 (3.12–5.83)	<0.01

Univariate analysis showed that there were significant differences between survivors and non-survivors regarding all three endpoints. Non-survivors were older (*p* < 0.01) and had higher pre-operative risk scores, as evidenced by higher EuroSCORE II values (*p* < 0.01 for all). The significant independent predictors of death included high BNP concentrations (*p* < 0.01), Troponin concentrations (*p* < 0.01), Creatinine concentrations (*p* < 0.01), and liver enzymes, such as ALT and AST (*p* < 0.01). The significant predictors included lower concentrations of Platelets (*p* < 0.01), Albumin (*p* ≤ 0.02), and GFR values (*p* < 0.01). Some of the significant associations included a lower incidence of smoking among non-survivors (*p* = 0.01, in-hospital). The significant surgical predictors included increased surgical time (*p* < 0.01, in-hospital) and increased incidence of mitral valve replacement surgery (*p* ≤ 0.02, all three).

### Feature selection and predictive model development

To analyze which features are most predictive for each mortality endpoint, the Boruta algorithm was used. The important features for in-hospital, 30-day, and 365-day mortalities are shown in Figure 1 (green). The important features can be seen to relate to cardiac function (features BNP and Troponin), renal function (features Creatinine and GFR), and liver function (features AST, ALT, and Bilirubin).

Afterwards, five separate prediction models were created and tested for validity: Logistic Regression, XGBoost, Random Forest, Extra Trees, and EuroSCORE II. The models’ performances are measured using various factors, which include Accuracy, Precision, Sensitivity, Specificity, and the Area under the Curve for the Receiver Operating Characteristics Curve (ROC AUC), and are seen in Tables 4–6. Visualizations using various graphs, including the ROC Curve, Precision-Recall Curve, and Decision Curve Analysis for every model, are shown in Figure 2. Supplementary Figures 1–3 provide additional visuals for each machine-learning model as well as additional calibration graphs.

**Table 4:** Predictive performance of five models (Logistic Regression, XGBoost, Random Forest, Extra Trees, and EuroSCORE II) for in-hospital mortality.

Model	Accuracy (95% CI)	Precision (95% CI)	Sensitivity (95% CI)	Specificity (95% CI)	ROC AUC (95% CI)	Average Precision (95% CI)
Logistic Regression	0.618 (0.568–0.809)	0.156 (0.100–0.286)	1.000 (0.840–1.000)	0.589 (0.537–0.804)	0.837 (0.761–0.906)	0.337 (0.158–0.560)
XGBoost	0.768 (0.647–0.925)	0.209 (0.124–0.474)	0.824 (0.579–1.000)	0.763 (0.628–0.937)	0.809 (0.669–0.931)	0.372 (0.198–0.627)
Random Forest	0.784 (0.743–0.896)	0.231 (0.145–0.409)	0.882 (0.680–1.000)	0.777 (0.729–0.905)	0.849 (0.738–0.940)	0.433 (0.239–0.655)
Extra Trees	0.784 (0.730–0.884)	0.231 (0.133–0.390)	0.882 (0.714–1.000)	0.777 (0.717–0.893)	0.858 (0.774–0.932)	0.342 (0.192–0.597)
EuroSCORE II	0.627 (0.560–0.693)	0.139 (0.073–0.210)	0.824 (0.630–1.000)	0.612 (0.545–0.683)	0.734 (0.603–0.857)	0.354 (0.148–0.578)

**Table 5:** Predictive performance of five models (Logistic Regression, XGBoost, Random Forest, Extra Trees, and EuroSCORE II) for 30-day mortality.

Model	Accuracy (95% CI)	Precision (95% CI)	Sensitivity (95% CI)	Specificity (95% CI)	ROC AUC (95% CI)	Average Precision (95% CI)
Logistic Regression	0.850 (0.433–0.917)	0.256 (0.085–0.469)	0.588 (0.533–1.000)	0.870 (0.395–0.941)	0.800 (0.688–0.900)	0.308 (0.148–0.574)
XGBoost	0.846 (0.575–0.921)	0.262 (0.094–0.458)	0.647 (0.455–1.000)	0.861 (0.552–0.944)	0.773 (0.639–0.897)	0.354 (0.148–0.573)
Random Forest	0.771 (0.496–0.929)	0.194 (0.098–0.500)	0.706 (0.444–1.000)	0.776 (0.471–0.951)	0.776 (0.637–0.899)	0.372 (0.161–0.582)
Extra Trees	0.796 (0.500–0.942)	0.214 (0.104–0.565)	0.706 (0.454–0.944)	0.803 (0.467–0.969)	0.777 (0.638–0.903)	0.399 (0.175–0.629)
EuroSCORE II	0.546 (0.483–0.604)	0.103 (0.055–0.164)	0.706 (0.467–0.938)	0.534 (0.465–0.600)	0.610 (0.470–0.752)	0.111 (0.062–0.238)

**Table 6:** Predictive performance of five models (Logistic Regression, XGBoost, Random Forest, Extra Trees, and EuroSCORE II) for 365-day mortality.

Model	Accuracy (95% CI)	Precision (95% CI)	Sensitivity (95% CI)	Specificity (95% CI)	ROC AUC (95% CI)	Average Precision (95% CI)
Logistic Regression	0.639 (0.600–0.926)	0.175 (0.120–0.559)	0.850 (0.455–1.000)	0.619 (0.580–0.953)	0.762 (0.639–0.869)	0.298 (0.147–0.503)
XGBoost	0.648 (0.591–0.861)	0.186 (0.117–0.308)	0.900 (0.650–1.000)	0.624 (0.561–0.882)	0.746 (0.633–0.846)	0.235 (0.131–0.442)
Random Forest	0.626 (0.496–0.887)	0.170 (0.109–0.400)	0.850 (0.545–1.000)	0.605 (0.454–0.915)	0.753 (0.634–0.851)	0.243 (0.127–0.452)
Extra Trees	0.574 (0.526–0.874)	0.158 (0.110–0.386)	0.900 (0.555–1.000)	0.543 (0.488–0.894)	0.772 (0.648–0.867)	0.328 (0.165–0.526)
EuroSCORE II	0.704 (0.643–0.757)	0.207 (0.122–0.291)	0.850 (0.688–1.000)	0.690 (0.628–0.748)	0.787 (0.692–0.873)	0.256 (0.150–0.465)

### Model performance and comparison

For predicting in-hospital mortality (Table 4), the machine learning models demonstrated strong discriminative performance. ROC-AUC values ranged from 0.809 (95% CI: 0.669–0.931) for XGBoost to 0.858 (95% CI: 0.774–0.932) for Extra Trees. The EuroSCORE II model yielded an accuracy of 0.627 (95% CI: 0.560–0.693) and specificity of 0.612 (95% CI: 0.545–0.683). Although its sensitivity was acceptable at 0.824 (95% CI: 0.630–1.000), overall discrimination remained inferior to several ML-based models. Pairwise statistical comparisons (Table 7) indicated that the Extra Trees model significantly outperformed EuroSCORE II (ROC-AUC: 0.858 vs. 0.734, *p* = 0.044).

**Table 7:** Pairwise comparisons of model discrimination for in-hospital mortality using DeLong’s test.

Model 1	Model 2	AUC 1	AUC 2	Delta AUC	Z-value	*P*-value
Extra Trees	EuroSCORE II	0.858	0.734	0.124	2.012	0.044
XGBoost	Random Forest	0.809	0.849	−0.040	−1.982	0.048
Random Forest	EuroSCORE II	0.849	0.734	0.114	1.790	0.074
XGBoost	Extra Trees	0.809	0.858	−0.049	−1.566	0.117
Logistic Regression	EuroSCORE II	0.837	0.734	0.103	1.535	0.125
XGBoost	EuroSCORE II	0.809	0.734	0.075	1.027	0.304
Logistic Regression	Extra Trees	0.837	0.858	−0.021	−0.797	0.426
Random Forest	Extra Trees	0.849	0.858	−0.009	−0.561	0.575
Logistic Regression	XGBoost	0.837	0.809	0.028	0.528	0.598
Logistic Regression	Random Forest	0.837	0.849	−0.012	−0.304	0.761

For predicting 30-day mortality (Table 5), Logistic Regression achieved the highest discrimination performance, with a ROC-AUC of 0.800 (95% CI: 0.688–0.900). The remaining machine learning models demonstrated comparable discriminatory capacity, with ROC-AUC values ranging from 0.773 (95% CI: 0.639–0.897) for XGBoost to 0.777 (95% CI: 0.638–0.903) for Extra Trees. In contrast, EuroSCORE II exhibited substantially lower discrimination, with a ROC-AUC of 0.610 (95% CI: 0.470–0.752). Logistic Regression yielded an accuracy of 0.850 (95% CI: 0.433–0.917), sensitivity of 0.588 (95% CI: 0.533–1.000), and specificity of 0.870 (95% CI: 0.395–0.941), indicating balanced classification performance. Pairwise statistical comparisons (Table 8) confirmed the superior discrimination performance of Logistic Regression relative to EuroSCORE II.

**Table 8:** Pairwise comparisons of model discrimination for 30-day mortality using DeLong’s test.

Model 1	Model 2	AUC 1	AUC 2	Delta AUC	Z-value	*P*-value
Logistic Regression	EuroSCORE II	0.800	0.610	0.190	2.205	0.027
XGBoost	EuroSCORE II	0.773	0.610	0.163	1.789	0.044
Extra Trees	EuroSCORE II	0.777	0.610	0.167	1.760	0.048
Random Forest	EuroSCORE II	0.776	0.610	0.166	1.739	0.082
Logistic Regression	Extra Trees	0.800	0.777	0.023	0.802	0.422
Logistic Regression	XGBoost	0.800	0.773	0.027	0.802	0.422
Logistic Regression	Random Forest	0.800	0.776	0.024	0.772	0.440
XGBoost	Extra Trees	0.773	0.777	−0.004	−0.191	0.849
XGBoost	Random Forest	0.773	0.776	−0.003	−0.169	0.866
Random Forest	Extra Trees	0.776	0.777	−0.001	−0.111	0.911

For predicting 365-day mortality (Table 6), all models demonstrated comparable discrimination performance. ROC-AUC values ranged from 0.746 (95% CI: 0.633–0.846) for XGBoost to 0.787 (95% CI: 0.692–0.873) for EuroSCORE II. EuroSCORE II achieved the highest ROC-AUC of 0.787 (95% CI: 0.692–0.873). The Extra Trees model demonstrated a similar ROC-AUC of 0.772 (95% CI: 0.648–0.867). Classification metrics were broadly balanced across models. EuroSCORE II yielded a sensitivity of 0.850 (95% CI: 0.688–1.000) and specificity of 0.690 (95% CI: 0.628–0.748). Pairwise comparisons of ROC-AUC values (Table 9) did not reveal statistically significant differences between models (all *p* > 0.05). As noted, these comparisons were exploratory and uncorrected for multiple testing.

**Table 9:** Pairwise comparisons of model discrimination for 365-day mortality using DeLong’s test.

Model 1	Model 2	AUC 1	AUC 2	Delta AUC	Z-value	*P*-value
XGBoost	Extra Trees	0.746	0.772	−0.026	−0.923	0.356
Random Forest	Extra Trees	0.753	0.772	−0.019	−0.879	0.380
XGBoost	EuroSCORE II	0.746	0.787	−0.040	−0.572	0.567
Random Forest	EuroSCORE II	0.753	0.787	−0.034	−0.480	0.631
Logistic Regression	XGBoost	0.762	0.746	0.016	0.411	0.681
XGBoost	Random Forest	0.746	0.753	−0.007	−0.337	0.736
Logistic Regression	EuroSCORE II	0.762	0.787	−0.025	−0.312	0.755
Logistic Regression	Extra Trees	0.762	0.772	−0.010	−0.297	0.767
Logistic Regression	Random Forest	0.762	0.753	0.009	0.208	0.836
Extra Trees	EuroSCORE II	0.772	0.787	−0.015	−0.200	0.841

### Nomogram construction and validation

**Figure 6:**
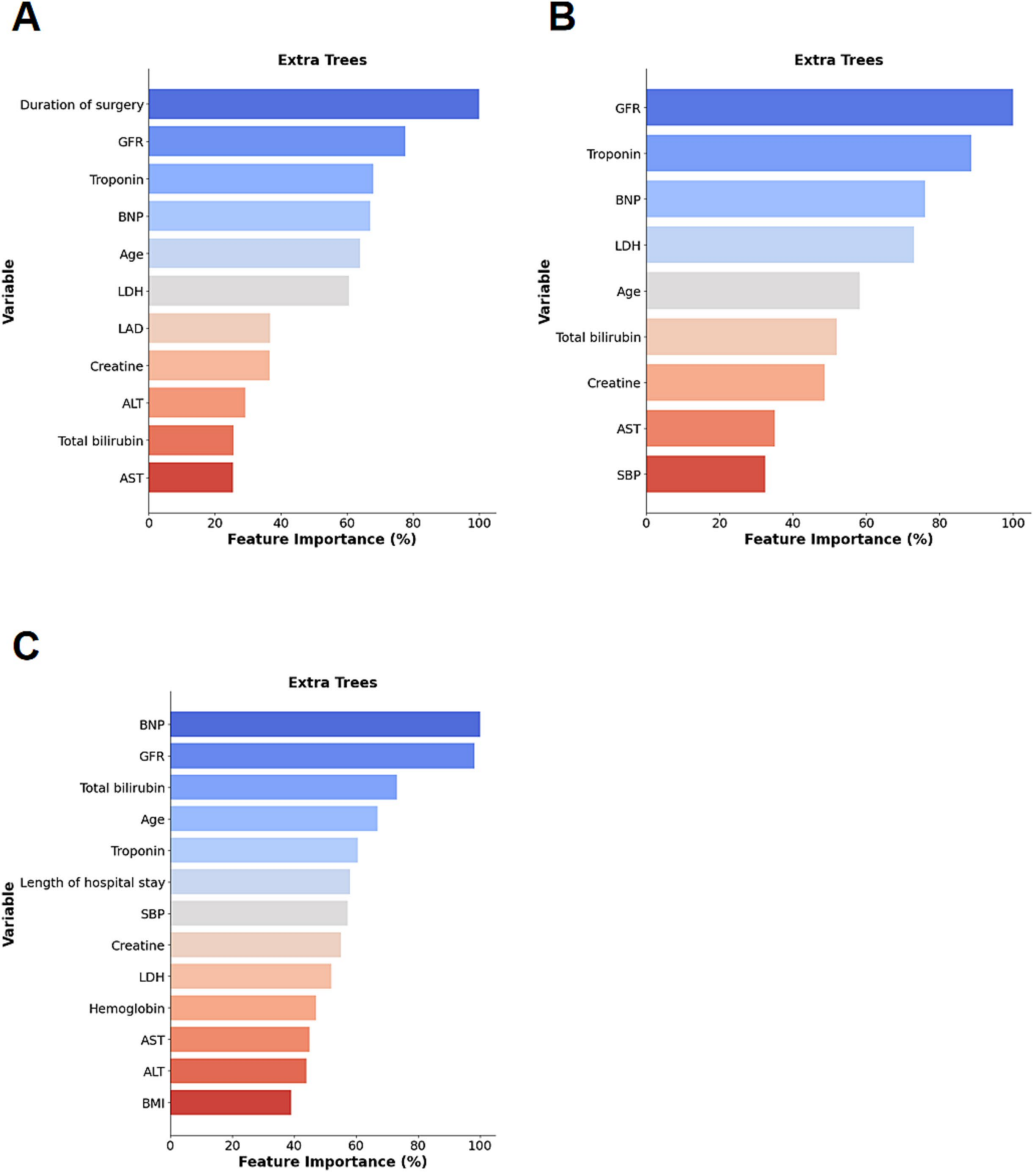
Feature importance rankings for the best-performing models across different prediction horizons: **(A)** top predictors for in-hospital mortality; **(B)** top predictors for 30-day mortality; **(C)** top predictors for 365-day mortality.

To enhance methodological consistency and clinical interpretability, predictor selection for nomogram development was guided by the feature importance derived from the Extra Trees models, as this model demonstrated superior and stable discrimination performance across prediction endpoints. The top contributing features identified by the Extra Trees models are presented in Figure 6. Across prediction tasks, key predictors consistently included age and biomarkers reflecting cardiac stress as well as renal and metabolic function. Predictors identified as clinically relevant were subsequently incorporated into a Logistic Regression framework for construction of interpretable nomograms. The final nomogram for predicting in-hospital, 30-day, and 365-day mortalities is shown in Figures 3A, 4A, 5A, respectively. The nomogram for predicting each endpoint was derived directly from its respective model. The nomograms performed well on the Validate Sets. The In-Hospital nomogram (Figures 3B,C) has good discriminative power and calibration. This also applied to the 30-day (Figures 4B,C) and 365-day (Figures 5B,C) models, which are well-calibrated and discriminative.

### External verification

In the external validation cohort consisting of 132 patients who underwent heart valve surgery, the models demonstrated consistent mortality risk prediction performance. For in-hospital mortality, the Extra Trees model achieved a ROC-AUC of 0.832 (95% CI: 0.741–0.909), with a sensitivity of 0.857 (95% CI: 0.667–0.952) and a specificity of 0.744 (95% CI: 0.681–0.843). For 30-day mortality, Logistic Regression yielded a ROC-AUC of 0.783 (95% CI: 0.688–0.900), with a sensitivity of 0.588 (95% CI: 0.533–1.000) and a specificity of 0.870 (95% CI: 0.395–0.941). For 365-day mortality, the Extra Trees model demonstrated a ROC-AUC of 0.772 (95% CI: 0.648–0.867), with a sensitivity of 0.900 (95% CI: 0.555–1.000) and a specificity of 0.543 (95% CI: 0.488–0.894). These findings indicate stable discrimination across short- and long-term outcomes (Supplementary Table 1).

## Discussion

In this multicenter retrospective cohort study, we evaluated and developed different Machine Learning algorithms for mortality rate estimation at different time points after heart valve operations. Our primary results clearly show that the Machine Learning methods, especially the ensemble algorithm Extra Trees, performed better on the in-hospital and 30-day mortality rate estimation tasks than the proven EuroSCORE II model. Additionally, we achieved the successful implementation of the developed models in the form of nomograms.

Our ML model’s better performance in terms of higher values for AUC of 0.858 for in-hospital mortality (Extra Trees) and 0.800 for 30-day mortality (Logistic Regression) is consistent with the increasing literature supporting the ability of ML to better perform compared to statistical models in cardiovascular outcomes. For instance, Mettananda et al. developed an ML-based model for cardiovascular risk in a Sri Lankan population and found that their model (AUC 0.72–0.74) was significantly better compared to the WHO risk chart (AUC 0.51) (15). Our results are also in line with another study by Bodenhofer et al. (16), focusing on 30-day mortality rate risk prediction after heart valve surgeries. Among their 2,229 studied patients, the Random Forest model showed significantly better performance compared to the EuroSCORE II model (AUC 0.839 compared to 0.704), respectively (16). This is consistent with our findings, where the performance gap between our ML models and the EuroSCORE II was most notable for 30-day mortality (AUC 0.800 vs. 0.610).

The use of these methodologies is also corroborated by the study of Jiang et al. (17), who developed a new predictive model for severe complications in patients having mitral valve surgery. Their XGBOOST algorithm attained a high level of performance (AUROC: 0.90) and highlighted 14 critical perioperative predictors, demonstrating the ability of these models in handling intricate clinical datasets. This trend of comparing sophisticated predictive methods with traditional predictive scores is likewise observed in other medical fields, as noted by Ye et al. (18) in their comparative analysis of nomograms and machine learning in the prediction of hematoma expansion in case of hypertensive intracerebral hemorrhage.

A notable observation from our analysis concerns the classification characteristics of EuroSCORE II for the 30-day mortality outcome. EuroSCORE II demonstrated modest discrimination performance, with a ROC-AUC of 0.610 (95% CI: 0.470–0.752). In terms of classification metrics, EuroSCORE II yielded a sensitivity of 0.706 (95% CI: 0.467–0.938) and a specificity of 0.534 (95% CI: 0.465–0.600). This pattern indicates limited discriminatory capacity relative to the machine learning models, which consistently achieved higher ROC-AUC values. However, in the context of pre-operational prognostic predictions for risk stratification in patient selection for interventions allocated prior to the procedure, no model predicting all patients to be at low risk is useful despite its high values of accuracy (19). Our ML models performed better by keeping high specificity values along with meaningful sensitivity values (e.g., 0.882 for in-hospital mortality and 0.870 for 30-day mortality), providing a useful prognostic tool for triggering interventions in high-risk patients.

The important characteristics discovered through the Boruta algorithm offer important pathophysiological clues, all of which seem intuitive and are supported in the general medical literature. The prominent role of markers of end-organ damage, namely high creatinine (renal), high AST/ALT, and thrombocytopaenia, underlines the fact that the risk of mortality post-operatively has implications beyond the domain of cardiac health alone. This subgroup has long supported the important role played by the patient’s residual physiology in determining surgical risk. Similar studies using nomograms in other diseases, including myeloma and post-operative pneumonia, have also named markers such as albumin, LDH, and creatinine, in all cases supporting the important general principle that general physiology has an important role in surgery (20, 21). The important role afforded in the model to BNP and Troponin re-emphasize the important role played by the patient’s pre-operative stress and injury burden in surgery (22). Enhanced model performance may be attributable to the incorporation of biomarkers not found in traditional surgical risk scores. Beyond the variables incorporated into the present models, cardiovascular risk evaluation in valve surgery commonly involves structural, functional, and rhythm-related assessments. Key cardiovascular domains relevant to surgical risk assessment are summarized in Supplementary Table 2. Due to the retrospective multicenter design, comprehensive imaging-derived parameters were not uniformly available across participating centers and therefore could not be reliably integrated into model development. Future studies incorporating standardized echocardiographic and imaging data may further refine predictive performance and enhance mechanistic interpretability. Although intraoperative variables may improve retrospective predictive accuracy, their inclusion may introduce post-treatment bias and substantially limit the clinical utility of models intended for preoperative risk assessment. Accordingly, predictor selection was deliberately restricted to simplified preoperative parameters that are readily accessible prior to surgery, thereby improving clinical usability, decision-making relevance, and potential bedside implementation.

In an attempt to fill the gap between the complex results provided by algorithmic models and the needs of practical application, we designed and validated nomograms using our most successful models. The process of developing a nomogram using an ML model specifically responds to the “black box” problem commonly encountered in the use of complex AI technology in the medical field and represents a valuable, accessible, and intuitive graphic aid for the individual risk prognosis and prediction in the medical field (20, 23). The high degree of “calibration” of our nomograms, illustrated in the corresponding figures, further underlines the valuable role that these tools could play in the reliable application process in the medical field and in ensuring the alignment between the model results and the real world, as reported in the pertinent discussion on nomograms in the field of hepatocellular carcinoma (21, 23). While ML models demonstrated superior predictive performance, several challenges must be considered when translating such approaches into clinical practice. Real-world clinical datasets are frequently affected by missing data, measurement variability, and heterogeneity in documentation standards, which may influence model robustness and external validity. Moreover, predictive accuracy alone does not guarantee clinical adoption. Interpretability, transparency, and clinician trust remain essential determinants of successful integration. Explainable AI techniques, including feature attribution methods, may enhance clinical interpretability by clarifying how individual predictors contribute to model outputs. Future research incorporating larger, standardized, and prospectively collected datasets, as well as hybrid modeling strategies combining statistical reasoning with machine learning flexibility, may further improve model reliability and clinical utility.

The strengths of our research include proper feature selection using Boruta, the ability to compare the performance of different models against EuroSCORE II, and the process of translating the model into nomograms. Nevertheless, several limitations should be considered. First, although external validation was conducted, both cohorts originated from centers within the same geographic region. Consequently, the generalizability and transportability of the models across different healthcare systems, patient populations, and institutional practices remain to be established. Variations in case-mix, institutional workflows, surgical volumes, and evolving treatment paradigms, including the increasing adoption of transcatheter interventions, may influence model performance in external clinical environments. Second, preoperative pharmacological variables were not incorporated. Future prospective investigations incorporating standardized medication data may further refine predictive performance and enhance clinical interpretability. Third, the relatively limited number of mortality events may introduce uncertainty in model stability, particularly for the 365-day outcome. Prediction models developed in low-event settings may be susceptible to performance variability despite internal and external validation safeguards. Therefore, results related to 365-day mortality should be interpreted with appropriate caution. Future studies using larger cohorts and prospectively collected datasets may further improve model stability and external validity. Finally, while the nomogram provides a clinically practical decision-support tool, prospective studies are necessary to determine its real-world clinical utility and potential influence on decision-making processes.

## Conclusion

In summary, the performance of our machine learning models was considerably better than the EuroSCORE II in the prediction of short-term mortality in individuals undergoing valve surgery, and the remarkably low sensitivity of the EuroSCORE II has major clinical limitations. The nomograms developed in this study provide a practical tool for individualized risk evaluation, with the potential to improve preoperative counseling and resource allocation for the high-risk population.

## Data Availability

The original contributions presented in the study are included in the article/Supplementary material, further inquiries can be directed to the corresponding authors.

## Supplementary material

SUPPLEMENTARY FIGURE 1 Calibration curves of the five prediction models for in-hospital mortality. **(A)** Logistic Regression. **(B)** XGBoost. **(C)** Random Forest. **(D)** Extra Trees. **(E)** EuroSCORE II.

SUPPLEMENTARY FIGURE 2 Calibration curves of the five prediction models for 30-day mortality. **(A)** Logistic Regression. **(B)** XGBoost. **(C)** Random Forest. **(D)** Extra Trees. **(E)** EuroSCORE II.

SUPPLEMENTARY FIGURE 3 Calibration curves of the five prediction models for 365-day mortality. **(A)** Logistic Regression. **(B)** XGBoost. **(C)** Random Forest. **(D)** Extra Trees. **(E)** EuroSCORE II.

Supplementary Table 1 Summary of external validation performance for the best-performing models across in-hospital, 30-day, and 365-day mortality.

Supplementary Table 2 Clinically relevant cardiovascular assessment domains in valve surgery risk evaluation.
