# Supplementary material for: Development and validation of machine learning nomograms for predicting mortality after cardiac valve surgery
Source: Front Med (Lausanne). 2026 Mar 27;13:1779140. doi: 10.3389/fmed.2026.1779140 (PMC13066181; doi:10.3389/fmed.2026.1779140)
Supplement: SUPPLEMENTARY FIGURE 1 — Calibration curves of the five prediction models for in-hospital mortality. (A) Logistic Regression. (B) XGBoost. (C) Random Forest. (D) Extra Trees. (E) EuroSCORE II. [file Presentation_1.pptx]

## Slide 1
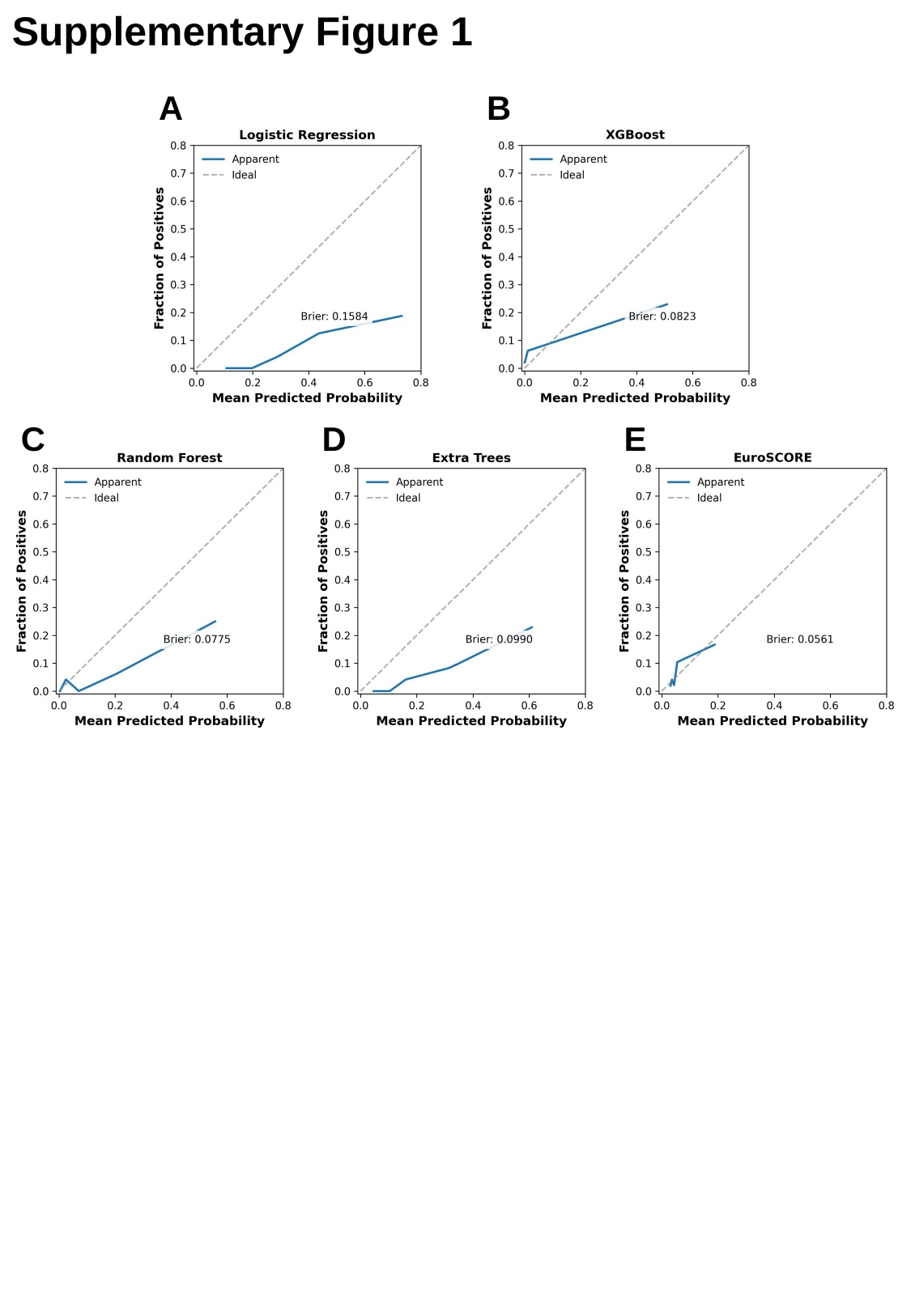

Supplementary Figure 1
B
A
D
E
C

## Slide 2
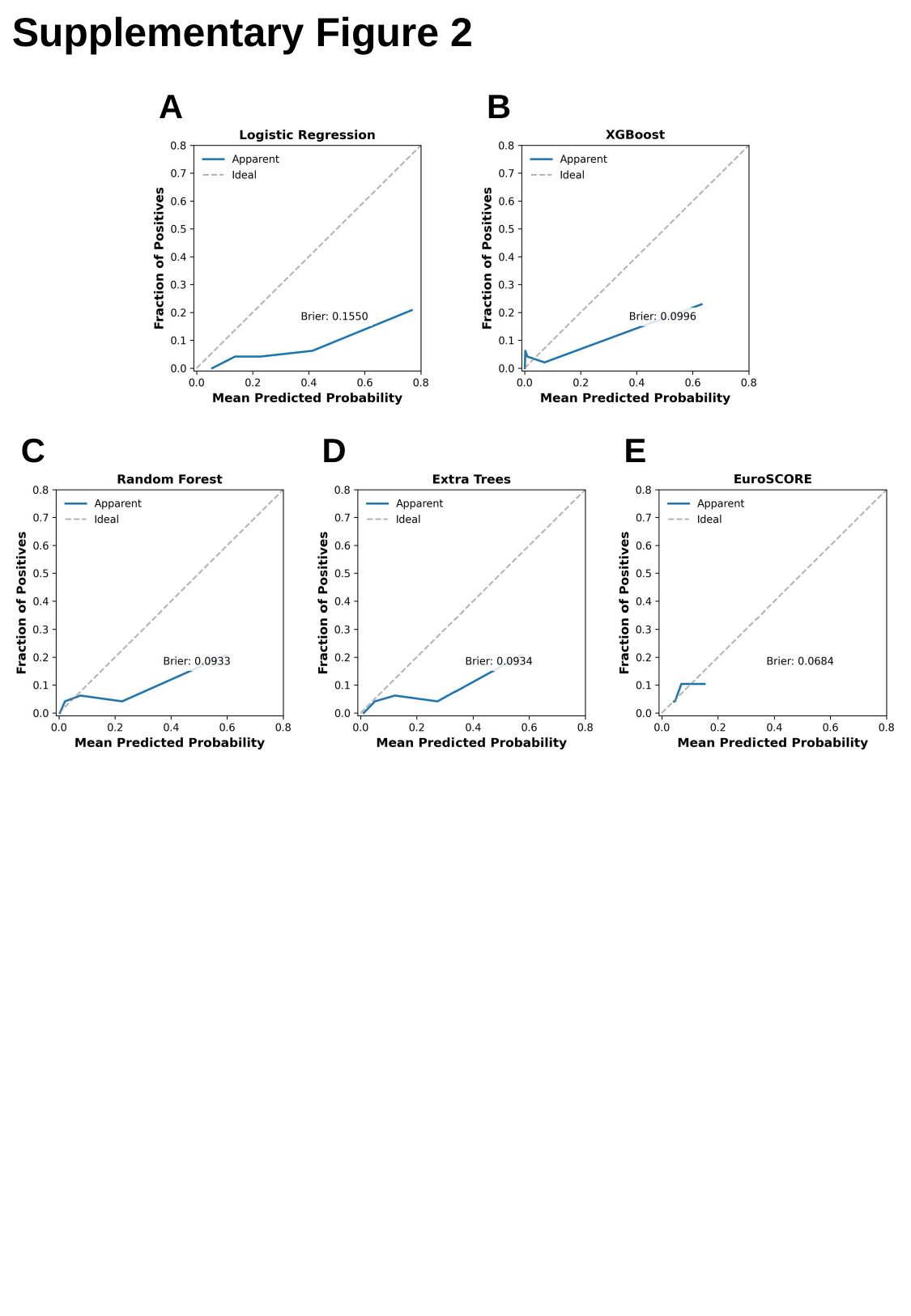

Supplementary Figure 2
B
A
D
E
C

## Slide 3
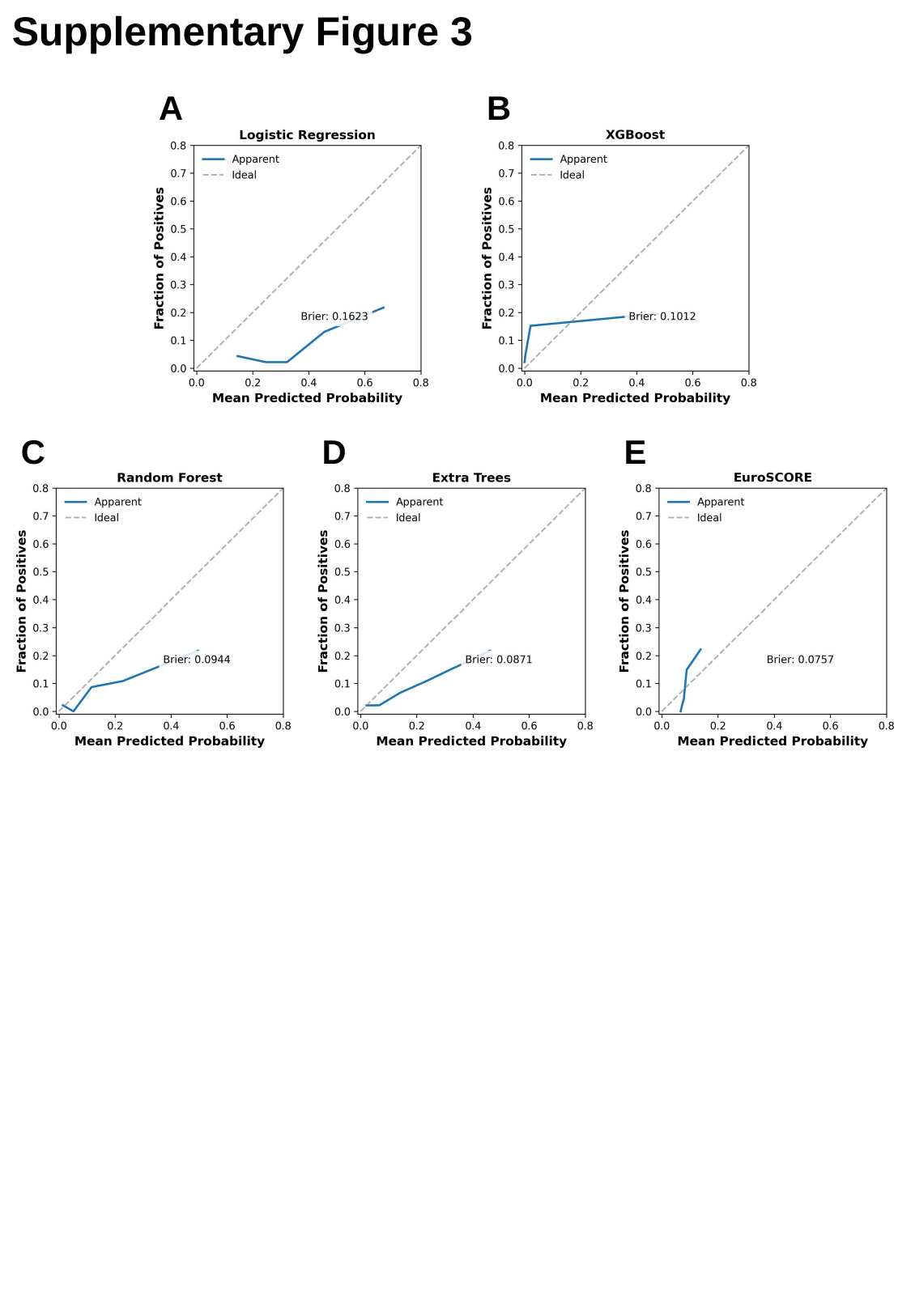

Supplementary Figure 3
B
A
D
E
C
