# Supplementary material for: Development and validation of machine learning nomograms for predicting mortality after cardiac valve surgery
Source: Front Med (Lausanne). 2026 Mar 27;13:1779140. doi: 10.3389/fmed.2026.1779140 (PMC13066181; doi:10.3389/fmed.2026.1779140)
Supplement: Supplementary Table 1 — Summary of external validation performance for the best-performing models across in-hospital, 30-day, and 365-day mortality. [file Table_1.docx]

| **Mortality** | **Model** | **Accuracy (95% CI)** | **Precision (95% CI)** | **Sensitivity (95% CI)** | **Specificity (95% CI)** | **ROC AUC (95% CI)** | **Average Precision (95% CI)** |
| --- | --- | --- | --- | --- | --- | --- | --- |
| In-hospital | Extra trees | 0.752 (0.690–0.812) | 0.248 (0.142–0.387) | 0.857 (0.667–0.952) | 0.744 (0.681–0.843) | 0.832 (0.741–0.909) | 0.366 (0.214–0.572) |
| 30-day | Logistic Regression | 0.850 (0.433–0.917) | 0.256 (0.085–0.469) | 0.588 (0.533–1.000) | 0.870 (0.395–0.941) | 0.783 (0.688–0.900) | 0.308 (0.148–0.574) |
| 365-day | Extra Trees | 0.574 (0.526–0.874) | 0.158 (0.110–0.386) | 0.900 (0.555–1.000) | 0.543 (0.488–0.894) | 0.772 (0.648–0.867) | 0.328 (0.165–0.526) |

**Supplementary Table 1.** Summary of external validation performance for the best-performing models across in-hospital, 30-day, and 365-day mortality.
