# Supplementary material for: Development and validation of machine learning nomograms for predicting mortality after cardiac valve surgery
Source: Front Med (Lausanne). 2026 Mar 27;13:1779140. doi: 10.3389/fmed.2026.1779140 (PMC13066181; doi:10.3389/fmed.2026.1779140)
Supplement: Supplementary Table 2 — Clinically relevant cardiovascular assessment domains in valve surgery risk evaluation. [file Table_2.docx]

**Supplementary Table 2.** Clinically relevant cardiovascular assessment domains in valve surgery risk evaluation.

| **Domain** | **Representative Variables** | **Clinical Relevance** |
| --- | --- | --- |
| **Left ventricular systolic function** | LVEF, LVESV, LVEDV | Reflect myocardial contractility and remodeling |
| **Left ventricular remodeling** | LV dilation / hypertrophy | Associated with chronic volume/pressure overload |
| **Right ventricular function** | TAPSE, RVFAC, RVSWI | Strong predictor of surgical outcomes |
| **Pulmonary circulation** | Pulmonary artery pressure | Reflect RV afterload and disease severity |
| **Right-sided filling pressures** | RAP, IVC indices | Hemodynamic burden |
| **Rhythm status** | Atrial fibrillation, conduction abnormalities | Influence perioperative risk |
| **Myocardial stress markers** | BNP / NT-proBNP | Ventricular strain & HF severity |
| **Valve morphology & severity** | Echo / CT findings | Structural assessment |
| **Advanced myocardial characterization** | Cardiac MRI (fibrosis / volumes) | Risk stratification |
